# Computing Ka and Ks with a consideration of unequal transitional substitutions

**DOI:** 10.1186/1471-2148-6-44

**Published:** 2006-06-02

**Authors:** Zhang Zhang, Jun Li, Jun Yu

**Affiliations:** 1 Institute of Computing Technology, Chinese Academy of Sciences, Beijing 100080, China; 2 Beijing Genomics Institute, Chinese Academy of Sciences, Beijing 101300, China; 3 Graduate School of Chinese Academy of Sciences, Beijing 100039, China; 4 James D. Watson Institute of Genome Sciences of Zhejiang University, Hangzhou Genomics Institute, Key Laboratory of Genomic Bioinformatics of Zhejiang Province, Hangzhou 310007, China

## Abstract

**Background:**

Approximate methods for estimating nonsynonymous and synonymous substitution rates (Ka and Ks) among protein-coding sequences have adopted different mutation (substitution) models. In the past two decades, several methods have been proposed but they have not considered unequal transitional substitutions (between the two purines, A and G, or the two pyrimidines, T and C) that become apparent when sequences data to be compared are vast and significantly diverged.

**Results:**

We propose a new method (MYN), a modified version of the Yang-Nielsen algorithm (YN), for evolutionary analysis of protein-coding sequences in general. MYN adopts the Tamura-Nei Model that considers the difference among rates of transitional and transversional substitutions as well as factors in codon frequency bias. We evaluate the performance of MYN by comparing to other methods, especially to YN, and to show that MYN has minimal deviations when parameters vary within normal ranges defined by empirical data.

**Conclusion:**

Our comparative results deriving from consistency analysis, computer simulations and authentic datasets, indicate that ignoring unequal transitional rates may lead to serious biases and that MYN performs well in most of the tested cases. These results also suggest that acquisitions of reliable synonymous and nonsynonymous substitution rates primarily depend on less biased estimates of transition/transversion rate ratio.

## Background

For appraising evolutionary significance of variable protein-coding sequences among diverged species in a quantitative fashion, one of the powerful tools is to compute nonsynonymous and synonymous substitution rates, termed as Ka and Ks, respectively [1-3]. Since Ka and Ks represent the numbers of substitutions per nonsynonymous and synonymous site, respectively, these parameters (or often their ratio ω = Ka/Ks) are used to partition the targeted sequences into three basic scenarios: negative (purifying) selection when Ka < Ks (ω < 1), positive (adaptive) selection when Ka > Ks (ω > 1), and neutral mutation when Ka = Ks (ω = 1).

Approximate methods for estimating Ka and Ks normally involve three steps: numbering synonymous (S) and nonsynonymous (N) sites, counting synonymous (S_d_) and nonsynonymous (N_d_) substitutions, and correcting for multiple substitutions. Over the past two decades, several methods have been developed [4-13], which are based on different mutation (substitution) models with subtle yet significant differences [14-16]. Among them, Yang and Nielsen made a valuable attempt to consider differences among transitional and transversional substitutions as well as codon frequency bias [4], and their method (denoted as YN in this report), based on the more realistic HKY Model [15] and implemented in PAML (Phylogenetic Analysis by Maximum Likelihood; [17]), has become increasingly popular in the field of molecular evolution studies. However, it does not exclude the possibility that methods based on simpler models have some favorable properties [18]. There are always tradeoffs between incorporating more features into models and avoiding over-parameterization for more accurate captures of evolutionary information [19-21]. In this report, we propose an improved method, a *m*odified *YN *algorithm or MYN, based on the Tamura-Nei Model, in which transitional changes (between A and G or T and C) are not assumed to occur with an equal frequency [22]. We also review the basics involved in estimating Ka and Ks (Table 1), explain differences between the two methods, and provide our comparative results as an in-depth evaluation for the new method.

**Table 1:** Symbols used in estimating Ka and Ks

Symbol	Definition
S	Number of synonymous sites
N	Number of nonsynonymous sites
S_d_	Number of synonymous substitutions
N_d_	Number of nonsynonymous substitutions
Ks	Synonymous substitution rate
Ka	Nonsynonymous substitution rate
ω	Estimator of selective strength, ω = Ka/Ks
*t*	Divergence time between two sequences, the expected number of nucleotide substitutions per codon, *t *= (Ks × 3S + Ka × 3N)/(S + N)
α_R_	Transitional rate between purines
α_Y_	Transitional rate between pyrimidines
α	Transitional rate
β	Transversional rate
κ_R_	Ratio of transitional rate between purines to transversional rate, κ_R _= α_R_/β
κ_Y_	Ratio of transitional rate between pyrimidines to transversional rate, κ_Y _= α_Y_/β
κ	Ratio of transitional rate/transversional rate, κ = α/β
*g*_N_	Frequency of nucleotide N, N ∈ {T, C, A, G}
π_j_	Frequency of codon *j*, *j *∈ {XYZ | X, Y, Z ∈ {T, C, A, G}}

## Results

To compare the performance of YN and MYN, we need to generate simulated and empirical datasets with careful considerations on possible features in evolution of diverged protein-coding sequences, such as biases in transitional rates and codon frequencies. We simulated hypothetical common ancestral sequences according to codon frequencies that were derived from three basic datasets: (1) equal codon frequencies (each sense codon frequency for canonical genetic code is 1/61, and other codes can be accommodated by making simple modifications [4]), (2) human codon frequencies (based on 39,420 human protein-coding genes from ENSEMBL database, Release 35 [23]), and (3) rice codon frequencies (deduced from 19,079 rice protein-coding genes [24]). We used the formula 100% × [(estimated value) - (expected value)]/(expected value) to calculate percentage errors for assessing relative biases between estimated and expected values.

### Consistency analysis

**Figure 1:**
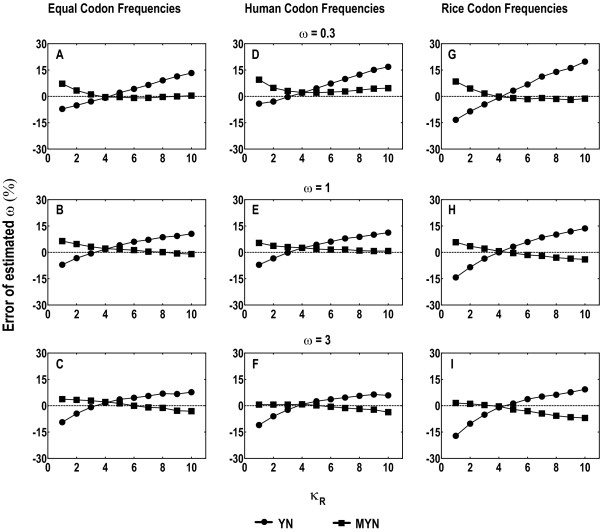
**Percentage errors of estimated ω (= Ka/Ks) by YN and MYN when κ_Y _= 3.75, considering κ_R _varying from 1 to 10**. The percentage error was calculated by the formula 100% × [(estimated value) - (expected value)]/(expected value). The canonical genetic code was used for simulated sequences with 2 million codons. Three sets of codon frequencies were used: equal (A to C), human (D to F) calculated from human protein-coding genes, and rice (G to I) calculated from rice protein-coding genes. ω = 0.3 (A, D, G), ω = 1 (B, E, H), and ω = 3 (C, F, I) were considered as representative values for purifying selection, neutral mutation and positive selection, respectively.

We first used a set of data that collectively contain 2 million codons, assuming that a good approximate method should not deviate too far from the real value with near infinite amount of data [4]. Although the selective strength, reflected in ω, differs from gene to gene, some representative values can be set based on analyses of empirical data; we use ω = 0.3, 1, and 3 as such values for negative, neutral, and positive mutations, respectively [3,4,25]. We fix *t *= 0.6 for the initial analysis and the effect of *t *is examined later. Since genuine values for κ often range from 1.5 to 5, we take 3.75 as a representative one. Considering that MYN differentiates κ_Y _from κ_R_, we always fix one of them to 3.75 and allow the other to vary from 1 to 10. We plotted percentage errors for ω between data generated with YN and MYN against κ_R _(fixing κ_Y _= 3.75) for different expected values, using the three codon frequencies from our test datasets (Figure 1 A to I). Similar results are readily obtained for fixed κ_R _and variable κ_Y _(data not shown).

Different codon frequencies have minor influence on the performance of YN and MYN. Ignoring the difference between κ_R _and κ_Y_, YN gives estimates close to the expected values only if κ_R _≈ κ_Y_. For instance, when κ_R _= 4, the percentage errors for ω calculated with YN under human codon frequencies are 1.98%, 2.51%, and 0.78% when ω = 0.3, 1, and 3, respectively (Figure 1D to 1F). When κ_R _≠ κ_Y_, YN gives rise to obviously biased ω; it tend to underestimate ω when κ_R _< κ_Y _and to overestimate ω when κ_R _> κ_Y_. The percentage errors for ω = 0.3, 1, and 3, when human codon frequencies are used, are -4.16%, -7.09% and -11.03% when κ_R _= 1, and are 16.82%, 11.24%, and 5.87% when κ_R _= 10, respectively. Compared to YN, MYN appears to produce lower percentage errors in ω estimations in most cases. When κ_R _≈ κ_Y_, MYN performs in a similar way as YN (it becomes equivalent to YN when κ_R _= κ_Y_).

**Figure 2:**
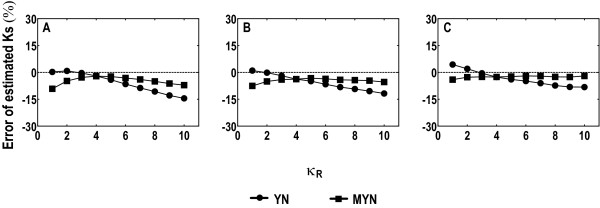
**Percentage errors of estimated Ks by YN and MYN when κ_Y _= 3.75, considering κ_R _varying from 1 to 10**. The percentage error was calculated by the formula 100% × [(estimated value) - (expected value)]/(expected value). Sequences with 2 million codons were simulated with human codon frequencies. Three representative values of ω (0.3 in A, 1 in B, and 3 in C) were used for purifying selection, neutral mutation, and positive selection, respectively.

We also compared YN and MYN in Ks estimations. We took a similar approach as what Tzeng and co-workers used to evaluate the expected values of Ks [12]. We plotted percentage errors of Ks against κ_R _(assuming κ_Y _= 3.75; Figure 2A to 2C) based on human codon frequencies, showing similar results when taking equal or rice codon frequencies (data not shown). YN and MYN give similar estimates of Ks when κ_R _≈ κ_Y_. When κ_R _= 4, the percentage errors of the estimated Ks generated by YN and MYN are -1.83% and -2.11% for ω = 0.3, -3.77% and -3.78% for ω = 1, -2.71% and -2.56% for ω = 3, respectively. YN tends to overestimate Ks when κ_R _< κ_Y _and to underestimate Ks when κ_R _> κ_Y _and the bias becomes serious with increasing κ_R_. MYN sometimes gives rise to larger biases than YN when κ_R _< κ_Y_, but it overall performs better for most of the parameter combinations tested, especially when κ_R _> κ_Y_.

### Effects of κ_R _and κ_Y_

We examined the effects of κ_R _and κ_Y _with a set of simulated sequences, three ω values (0.3, 1 and 3), and two *t *values (0.1 and 1). We considered the case of κ_R _≥ κ_Y _and four parameter sets: (1) κ_R _= 3 and κ_Y _= 1.5, (2) κ_R _= 5 and κ_Y _= 1.5, (3) κ_R _= 10 and κ_Y _= 1, (4) κ_R _= κ_Y _= 3.75. To avoid stochastic errors, we generated 1,000 pairs of sequences with 400 codons each for all the tests. We only described the results when human codon frequencies were used since similar results were obtained for equal or rice codon frequencies. The average estimates of Ka, Ks, and ω were computed with YN and MYN as well as expected values of Ka and Ks (Table 2).

**Table 2:** Average estimates of Ka, Ks, and ω with YN and MYN

Parameters				Expected Values		YN			MYN		
			
ω	*t*	κ_R_	κ_Y_	Ka	Ks	Ka	Ks	ω	Ka	Ks	ω
0.3	0.1	3	1.5	0.021	0.069	0.021	0.065	0.353	0.021	0.067	0.340
		5	1.5	0.021	0.069	0.021	0.062	0.361	0.021	0.066	0.332
		10	1	0.020	0.068	0.022	0.058	0.395	0.021	0.066	0.334
		3.75	3.75	0.020	0.066	0.020	0.066	0.328	0.020	0.066	0.331
	1	3	1.5	0.207	0.692	0.210	0.653	0.329	0.208	0.720	0.298
		5	1.5	0.206	0.686	0.206	0.569	0.369	0.201	0.672	0.311
		10	1	0.205	0.682	0.197	0.419	0.476	0.188	0.529	0.366
		3.75	3.75	0.199	0.662	0.198	0.662	0.305	0.198	0.676	0.301
											
1	0.1	3	1.5	0.033	0.033	0.034	0.032	1.216	0.034	0.033	1.163
		5	1.5	0.033	0.033	0.034	0.030	1.294	0.034	0.032	1.187
		10	1	0.033	0.033	0.034	0.030	1.293	0.033	0.033	1.102
		3.75	3.75	0.033	0.033	0.034	0.033	1.144	0.034	0.033	1.150
	1	3	1.5	0.333	0.333	0.330	0.305	1.103	0.325	0.322	1.034
		5	1.5	0.333	0.333	0.325	0.283	1.168	0.317	0.310	1.044
		10	1	0.333	0.333	0.300	0.242	1.267	0.287	0.279	1.051
		3.75	3.75	0.333	0.333	0.326	0.318	1.043	0.327	0.318	1.047
											
3	0.1	3	1.5	0.040	0.013	0.041	0.013	3.637	0.040	0.014	3.511
		5	1.5	0.041	0.014	0.041	0.013	3.738	0.040	0.014	3.453
		10	1	0.041	0.014	0.041	0.014	3.077	0.039	0.016	2.783
		3.75	3.75	0.041	0.014	0.040	0.016	2.846	0.041	0.016	2.869
	1	3	1.5	0.403	0.134	0.396	0.129	3.173	0.391	0.135	2.994
		5	1.5	0.405	0.135	0.389	0.122	3.304	0.379	0.132	2.986
		10	1	0.406	0.135	0.354	0.113	3.216	0.340	0.128	2.734
		3.75	3.75	0.413	0.138	0.400	0.136	3.015	0.402	0.136	3.026

Note: The values were averaged over 1,000 pairs of simulated sequences that each had 400 codons.

Without considering the difference between κ_R _and κ_Y_, YN produces minor bias when κ_R _= κ_Y_, and underestimates Ks and overestimates ω when κ_R _> κ_Y_. MYN is less biased compared with YN for most parameter combinations. The results agree with the infinite data test for consistency. For example, when κ_R _> κ_Y_, YN gives positive values for ω and negative values for Ks in percentage errors for the infinite data. As a result, it overestimates ω and underestimates Ks for sequences with normal length such as 400 codons albeit far from dramatic.

### Effect of *t*

**Figure 3:**
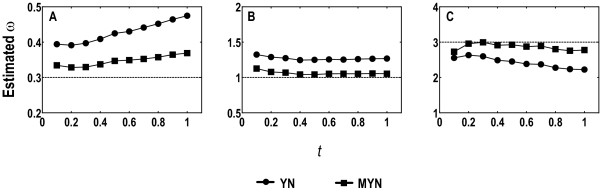
**Average ω estimates over 1,000 pairs of sequences when divergence time *t *varies from 0.1 to 1**. Human codon frequencies were used for simulating sequences (400 codons each). The parameters are ω = 0.3, κ_R _= 10, κ_Y _= 1 in A; ω = 1, κ_R _= 10, κ_Y _= 1 in B; and ω = 3, κ_R _= 1, κ_Y _= 10 in C. Note that the scales of y-axis are different in all three panels.

We let *t *vary from 0.1 to 1 to evaluate its effect. We again used the human codon frequencies for simulations (1,000 pairs of sequences with 400 codons for each case), and tested three different parameter combinations: (1) ω = 0.3, κ_R _= 10 and κ_Y _= 1; (2) ω = 1, κ_R _= 10 and κ_Y _= 1; (3) ω = 3, κ_Y _= 10 and κ_R _= 1. We plotted average estimates of ω with YN and MYN against *t *for the parameter combinations (Figure 3A to 3C).

Both YN and MYN have a nearly parallel overall trend when *t *varies from 0.1 to 1 but MYN deviates less from the expected values. They both tend to overestimate ω for purifying selection and to underestimate ω for positive selection, whereas *t *has little influence on ω for neutral mutation. YN overestimates ω when κ_R _> κ_Y _and underestimates ω when κ_R _< κ_Y_, which is consistent with those found in the infinite data test. MYN tends to overestimate ω for the expected ω = 0.3 and this overestimation becomes severe with increasing *t*, but it is relatively subtle when compared to YN. When ω = 1 and 3, MYN gives closer ω estimates than YN over most of the parameter combinations.

### Effect of sequence length

To examine the effect of variable protein length (number of codons), we took the human codon frequencies, 1,000 pairs of sequences for each case, and two sets of parameters for the analysis: (1) ω = 0.3, κ_R _= 10, κ_Y _= 1, and *t *= 1; (2) ω = 3, κ_R _= 1, κ_Y _= 10, and *t *= 0.1. We calculated the average ω estimates when numbers of codons vary from 100 to 1,000 for the simulated sequences (Table 3). YN and MYN both give larger biases for short sequences (< 300 codons). Despite the fact that YN and MYN do not perform satisfactorily when target sequences are rather short, MYN is less biased than YN for most of the parameter settings.

**Table 3:** Average estimates of ω with YN and MYN

Number of codons	ω = 0.3		ω = 3	
		
	YN	MYN	YN	MYN
100	0.504	0.408	2.092	2.014
200	0.477	0.369	2.340	2.394
300	0.473	0.368	2.522	2.627
400	0.473	0.364	2.583	2.736
500	0.470	0.363	2.568	2.824
600	0.469	0.360	2.661	2.929
700	0.472	0.363	2.653	2.943
800	0.469	0.361	2.684	3.003
900	0.466	0.358	2.695	3.034
1000	0.473	0.361	2.671	3.006

Note: The parameters used were κ_R _= 10, κ_Y _= 1 and *t *= 1 for purifying selection (ω = 0.3), and κ_R _= 1, κ_Y _= 10, and *t *= 0.1 for positive selection (ω = 3). The ω values were averaged over 1,000 pairs of simulated sequences.

### Testing real data

**Figure 4:**
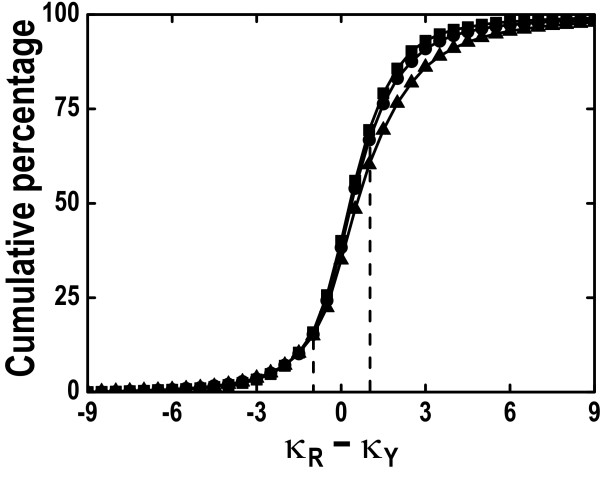
**Cumulative percentage of κ_R _– κ_Y _for human-mouse (squares), human-dog (circles), and mouse-rat (triangles) orthologs at a bin size of 0.1**. Dashed lines were used to show the cases when κ_R_-κ_Y _= -1 and κ_R_-κ_Y _= 1.

We collected three orthologous datasets from NCBI HomoloGene database (Build 44.1 [26]): 14,329 pairs of human-mouse, 10,851 pairs of human-dog, and 13,544 pairs of mouse-rat. For a more comprehensive display, we examined the cumulative percentage of κ_R _- κ_Y _(Figure 4), emphasizing different transitional substitutions with unequal frequencies. For instance, the cumulative percentages for κ_R _- κ_Y _> 1 for human-mouse, human-dog, and mouse-rat orthologs are 30.6%, 33.2%, and 39.7%, respectively, and those for κ_R _- κ_Y _< -1 are all approximately 15%. The rest, for | κ_R _- κ_Y _| ≤ 1, are 53.6%, 51.5%, and 45.4% for the three ortholog groups, respectively.

To evaluate the performance of MYN, we compared a set of values (S%, Ka, Ks, and ω) generated with three other selected methods in a similar way, considering κ_R _- κ_Y _> 1, κ_R _- κ_Y _< -1, and | κ_R _- κ_Y _| ≤ 1 (Table 4). Other than YN, we used another approximate method developed by Li (1993) and by Pamilo and Bianchi (1993) independently (denoted as LPB), and a maximum likelihood method proposed by Goldman and Yang (1994) (denoted as GY). Despite the fact that all four methods yield similar Ka estimates, they do show some differences in other parameter settings. As a whole, LPB tends to overestimate ω and to underestimate Ks when compared to MYN. GY performs similarly as YN does since both consider transition/transversion rate bias and nucleotide (codon) frequency bias. For instance, if κ_R _- κ_Y _< -1, both methods, compared to MYN, underestimate S% and ω, and overestimate Ks as we have demonstrated in the simulation studies. In the case of | κ_R _- κ_Y _| ≤ 1, YN and MYN work in a similar way. When confined to κ_R _- κ_Y _> 1, GY and YN both overestimate S% and ω, and underestimate Ks, compared to MYN. Taking the Ks estimates as an example, they are 0.527 and 0.597 for human-mouse orthologs, 0.329 and 0.357 for human-dog orthologs, and 0.186 and 0.201 for mouse-rat orthologs, calculated with YN and MYN, respectively. Similarly, the estimated ω with YN and MYN are 0.121 and 0.105 for human-mouse orthologs, 0.158 and 0.143 for human-dog orthologs, and 0.157 and 0.142 for mouse-rat orthologs, respectively.

**Table 4:** Proportions of synonymous sites (S%) and estimates of Ka, Ks and ω

Method	κ_R _– κ_Y _> 1				κ_R _– κ_Y _< -1				|κ_R _– κ_Y_|≤ 1			
			
	S%	Ka	Ks	ω	S%	Ka	Ks	ω	S%	Ka	Ks	ω
human-mouse orthologs												
LPB	-	0.069	0.463	0.148	-	0.071	0.449	0.159	-	0.105	0.500	0.209
GY	27.2%	0.065	0.518	0.125	27.1%	0.068	0.503	0.135	26.9%	0.101	0.561	0.180
YN	27.4%	0.064	0.527	0.121	27.2%	0.067	0.505	0.133	26.6%	0.099	0.588	0.169
MYN	26.1%	0.063	0.597	0.105	28.5%	0.068	0.474	0.144	26.5%	0.099	0.591	0.168
												
human-dog orthologs												
LPB	-	0.055	0.309	0.176	-	0.057	0.296	0.192	-	0.081	0.348	0.233
GY	27.5%	0.052	0.332	0.157	27.5%	0.055	0.318	0.172	26.5%	0.078	0.381	0.205
YN	27.8%	0.052	0.329	0.158	27.8%	0.055	0.310	0.176	26.4%	0.077	0.387	0.200
MYN	26.5%	0.051	0.357	0.143	29.1%	0.056	0.294	0.189	26.3%	0.077	0.389	0.199
												
mouse-rat orthologs												
LPB	-	0.030	0.176	0.173	-	0.030	0.170	0.179	-	0.048	0.196	0.245
GY	28.0%	0.030	0.189	0.157	27.7%	0.029	0.183	0.160	26.9%	0.047	0.214	0.220
YN	28.2%	0.029	0.186	0.157	27.9%	0.029	0.180	0.162	26.6%	0.046	0.216	0.215
MYN	26.6%	0.029	0.201	0.142	29.1%	0.030	0.171	0.173	26.5%	0.046	0.216	0.214

Note: The values were calculated from concatenated sequences according to the three scenarios of κ_R _and κ_Y_. The methods compared in addition to YN and MYN were LPB, an approximate method developed by Li (1993) and by Pamilo and Bianchi (1993) independently, and GY, a maximum likelihood method proposed by Goldman and Yang (1994). When GY was used, codon frequencies were calculated from nucleotide frequencies of three codon positions.

## Discussion

MYN, proposed as a modified YN in this paper, allows for unequal transitional rates between purines and between pyrimidines as well as transversional rate and nucleotide (codon) frequencies. Our comparative analyses indicate that ignoring unequal transitional rates often results in closer estimates only when κ_R _≈ κ_Y _but rather biased estimates when κ_R _> κ_Y _or κ_R _< κ_Y_, and that MYN is more robust in simulations and real datasets by comparison with other methods, especially with YN. Therefore, it is important to take account of unequal transitional rates for accurately capturing evolutionary information (see one of Ka and Ks applications; [27]) when unequal transitional rates among compared sequences exist. In addition, unequal transitional rates can also be implemented in a maximum likelihood framework [10] that allows model evaluation for choosing a better suit to a dataset and therefore allows users to obtain more reliable estimates.

### How does κ lead to biased estimates of Ka and Ks?

All approximate methods for Ka and Ks estimations may one way or another give rise to biased results for at least some parameter combinations. We examined κ, which is used not only in estimating S and N, but also in generating a transition probability matrix for estimating S_d _and N_d_. Since the sum of S_d _and N_d _between the two compared sequences is always smaller than the sum of possible sites (S + N or the effective length of compared sequences), the influence of κ on S and N is significantly stronger than that on S_d _and N_d_. Hence, let us focus on the effect of κ on S and N. YN uses κ and codon frequencies to estimate S and N. Since codon frequencies are constant, estimated from targeted sequences, and transitions between two codons are more likely to be synonymous especially at the third codon positions, S thus (as a function of κ and codon frequencies) is positively correlated to κ. Furthermore, the values of Ks depend on S (Ks ≈ S_d _/S, assuming no correction for multiple substitutions). Therefore, an overestimated κ could give rise to overestimation of S and underestimation of Ks, which results in overestimation of ω. Likewise, underestimation of κ leads to overestimation of Ks and underestimation of ω. Therefore, κ correlates negatively with Ks and positively with ω and such correlations are also applicable to κ_R _and κ_Y_.

Let us examine how κ affects Ka and Ks in our simulations. When κ_R _< κ_Y_, YN tends to underestimate ω and to overestimate Ks, which results from the underestimation of κ by assuming κ_Y _equal to κ_R_. The trend sometimes is less obvious, because about 4% loss of sites are due to mutations leading to stop codons, resulting in slightly underestimated Ka and Ks [4,28]. Similarly, when κ_R _> κ_Y_, YN overestimates κ, resulting in overestimation of ω and underestimation of Ks. The bias of ω becomes more pronounced as the bias of κ varies to extremes, when κ_R _>> κ_Y _or κ_R _<< κ_Y_. In addition, κ_R _and κ_Y _can also affect the estimates from MYN. Since transitions are more likely to occur than transversions, a decrease in κ_R _can be related to more drastic reduction of transversions than transitions between purines, resulting in overestimated κ_R_; this overestimation becomes severer for purifying selection due to a higher occurrence of synonymous substitutions than nonsynonymous ones, and synonymous substitutions are also more likely to be transitional. As a result, ω can be overestimated (Figure 1) and Ks can be underestimated (Figure 2), especially when κ_R _values are smaller. Therefore, to acquire more reliable estimates on Ka and Ks, it is essential to have a less biased estimate of κ, or both κ_R _and κ_Y_.

### Influences of *t *and sequence length

Divergence time (*t*) and sequence length both influence the estimation of Ka and Ks. Since κ in YN, κ_R _and κ_Y _in MYN are all deduced from fourfold-degenerate sites at the third codon positions and non-degenerate sites (often the first and the second codon positions), larger *t *leads to multiple substitutions between the two compared codons, resulting in under-counts of substitution events (a reduction in effective sampling size) for estimating κ, κ_R _and κ_Y_. Therefore, extreme *t *tends to decrease the effective sampling size and short sequences tend to exaggerate this effect, resulting in biased estimates of κ, κ_R _and κ_Y_. We indeed found that both YN and MYN tend to overestimate ω for purifying selection and underestimate ω for positive selection and these biases become severe as *t *increases (Figure 3). These biased estimations are in fact indirect results of biased estimates of κ, κ_R _and κ_Y_. The fact is that synonymous substitutions are more likely resulted from transitions than nonsynonymous ones are, such as the cases of two-fold degenerate codons, so the bias between κ_R _and κ_Y _does exist. In addition, since for negative selection, synonymous substitutions have higher possibilities to occur than nonsynonymous ones, transitions are more likely to occur than transversions. Therefore, with a decreasing effective sampling size caused by the increasing *t*, YN tends to underestimate transversional rate and thus to overestimate κ. In the case of positive selection, YN underestimates transitional rate and κ in a similar way. As to neutral mutation, YN tends to simultaneously underestimate both transitional and transversional rates so that the varying *t *has no apparent effect. Since MYN adopts a similar approach as YN estimates κ, it shows a similar trend. Short sequences have similar effect as smaller sampling size, and therefore, both YN and MYN may give rise to biased estimates κ, κ_R _and κ_Y_, albeit MYN's better performance in less extreme cases.

## Conclusion

We compared MYN with other methods, especially with YN, by examining infinitely long sequences, performing computer simulations and analyzing real datasets, and found that MYN has minimal deviation even when parameters vary within normal ranges defined by empirical data. In addition, these results indicate that biased estimates of Ka and Ks primarily stem from biased estimates of κ, or both κ_R _and κ_Y_, which can be influenced by *t *and sequence length.

## Methods

Methods for estimating Ka and Ks consider sequence variations of both DNA and protein, which are related through the genetic code. Since we are engaging in a generally purposed discussion, the genetic code is always referred to the canonical code. As a DNA-centric consideration, nucleotides substitutions only have two types, either within purines and pyrimidines as transitions or between them as transversions. As a protein-centric consideration, each nucleotide triplet (codon) is defined as they vary according to nucleotide changes, except stop codons (TAG, TAA, and TGA). For protein-coding genes, nucleotide substitutions are classified as nonsynonymous and synonymous (silent), referring to changes that do or do not provoke amino acid variations. Although there are several mutation (substitution) models that take these sequence variation features into account, in this report we limit our discussion only to the HKY and the Tamura-Nei Models (see Table S1 in the additional file 1 for details).

### Mutation model

YN adopts the HKY Model that considers transitional rate, transversional rate, and unequal nucleotide (codon) frequencies. It uses an iterative approach to estimate Ka and Ks. Before iteration, YN computes nucleotide frequencies (regarding to the three codon positions), κ, S and N from compared sequences. Codon frequencies are calculated by multiplying each nucleotide frequencies. κ is estimated from fourfold degenerate sites at the third codon positions and non-degenerate sites. S and N are calculated by using κ and codon frequencies. YN then chooses initial values for *t *and ω as starting point for iteration. It generates a transition probability matrix that represents substitution probabilities from one codon to another by using ω, *t*, κ, and codon frequencies. This transition probability matrix is then used to deduce S_d _and N_d_. Hence, new estimates of ω and *t *can be obtained. YN repeats the calculation for another transition probability matrix, until the algorithm converges.

Compared to the HKY Model, the Tamura-Nei Model simply considers more parameters. It differentiates α_R _from α_Y _according to different transitional substitutions. In fact, if the transitional rates between purines and between pyrimidines are set equal (α_R _= α_Y_), the model becomes the HKY Model. Since the Tamura-Nei Model distinguishes α_R _and α_Y_, we correspondingly use κ_R _and κ_Y _to denote the ratios of transitional rates between purines and between pyrimidines over the transversional rate, respectively. MYN also needs a transition probability matrix similar to what YN has. We give the substitution rate *q*_*ij *_from any sense codon *i *to *j *(*i *≠ *j*) to generate a transition probability matrix as follows:

qij={0,if i and j differ by more than one differnceπj,if i and j differ by a synonymous transversionκRπj,if i and j differ by a synonymous transition between purinesκYπj,if i and j differ by a synonymous transition between pyrimidinesωπj,if i and j differ by a nonsynonymous transversionωκRπj,if i and j differ by a nonsynonymous transition between purinesωκYπj,if i and j differ by a nonsynonymous transition between pyrimidines     (1)
 MathType@MTEF@5@5@+=feaafiart1ev1aaatCvAUfKttLearuWrP9MDH5MBPbIqV92AaeXatLxBI9gBaebbnrfifHhDYfgasaacH8akY=wiFfYdH8Gipec8Eeeu0xXdbba9frFj0=OqFfea0dXdd9vqai=hGuQ8kuc9pgc9s8qqaq=dirpe0xb9q8qiLsFr0=vr0=vr0dc8meaabaqaciaacaGaaeqabaqabeGadaaakeaacqWGXbqCdaWgaaWcbaGaemyAaKMaemOAaOgabeaakiabg2da9maaceaabaqbaeaabyqaaaaabaGaeGimaaJaeiilaWIaeeyAaKMaeeOzayMaeeiiaaIaemyAaKMaeeiiaaIaeeyyaeMaeeOBa4MaeeizaqMaeeiiaaIaemOAaOMaeeiiaaIaeeizaqMaeeyAaKMaeeOzayMaeeOzayMaeeyzauMaeeOCaiNaeeiiaaIaeeOyaiMaeeyEaKNaeeiiaaIaeeyBa0Maee4Ba8MaeeOCaiNaeeyzauMaeeiiaaIaeeiDaqNaeeiAaGMaeeyyaeMaeeOBa4MaeeiiaaIaee4Ba8MaeeOBa4MaeeyzauMaeeiiaaIaeeizaqMaeeyAaKMaeeOzayMaeeOzayMaeeyzauMaeeOCaiNaeeOBa4Maee4yamMaeeyzaugabaGaeqiWda3aaSbaaSqaaiabdQgaQbqabaGccqGGSaalcqqGPbqAcqqGMbGzcqqGGaaicqWGPbqAcqqGGaaicqqGHbqycqqGUbGBcqqGKbazcqqGGaaicqWGQbGAcqqGGaaicqqGKbazcqqGPbqAcqqGMbGzcqqGMbGzcqqGLbqzcqqGYbGCcqqGGaaicqqGIbGycqqG5bqEcqqGGaaicqqGHbqycqqGGaaicqqGZbWCcqqG5bqEcqqGUbGBcqqGVbWBcqqGUbGBcqqG5bqEcqqGTbqBcqqGVbWBcqqG1bqDcqqGZbWCcqqGGaaicqqG0baDcqqGYbGCcqqGHbqycqqGUbGBcqqGZbWCcqqG2bGDcqqGLbqzcqqGYbGCcqqGZbWCcqqGPbqAcqqGVbWBcqqGUbGBaeaacqaH6oWAdaWgaaWcbaGaeeOuaifabeaakiabec8aWnaaBaaaleaacqWGQbGAaeqaaOGaeiilaWIaeeyAaKMaeeOzayMaeeiiaaIaemyAaKMaeeiiaaIaeeyyaeMaeeOBa4MaeeizaqMaeeiiaaIaemOAaOMaeeiiaaIaeeizaqMaeeyAaKMaeeOzayMaeeOzayMaeeyzauMaeeOCaiNaeeiiaaIaeeOyaiMaeeyEaKNaeeiiaaIaeeyyaeMaeeiiaaIaee4CamNaeeyEaKNaeeOBa4Maee4Ba8MaeeOBa4MaeeyEaKNaeeyBa0Maee4Ba8MaeeyDauNaee4CamNaeeiiaaIaeeiDaqNaeeOCaiNaeeyyaeMaeeOBa4Maee4CamNaeeyAaKMaeeiDaqNaeeyAaKMaee4Ba8MaeeOBa4MaeeiiaaIaeeOyaiMaeeyzauMaeeiDaqNaee4DaCNaeeyzauMaeeyzauMaeeOBa4MaeeiiaaIaeeiCaaNaeeyDauNaeeOCaiNaeeyAaKMaeeOBa4MaeeyzauMaee4CamhabaGaeqOUdS2aaSbaaSqaaiabbMfazbqabaGccqaHapaCdaWgaaWcbaGaemOAaOgabeaakiabbYcaSiabbMgaPjabbAgaMjabbccaGiabdMgaPjabbccaGiabbggaHjabb6gaUjabbsgaKjabbccaGiabdQgaQjabbccaGiabbsgaKjabbMgaPjabbAgaMjabbAgaMjabbwgaLjabbkhaYjabbccaGiabbkgaIjabbMha5jabbccaGiabbggaHjabbccaGiabbohaZjabbMha5jabb6gaUjabb+gaVjabb6gaUjabbMha5jabb2gaTjabb+gaVjabbwha1jabbohaZjabbccaGiabbsha0jabbkhaYjabbggaHjabb6gaUjabbohaZjabbMgaPjabbsha0jabbMgaPjabb+gaVjabb6gaUjabbccaGiabbkgaIjabbwgaLjabbsha0jabbEha3jabbwgaLjabbwgaLjabb6gaUjabbccaGiabbchaWjabbMha5jabbkhaYjabbMgaPjabb2gaTjabbMgaPjabbsgaKjabbMgaPjabb6gaUjabbwgaLjabbohaZbqaaiabeM8a3jabec8aWnaaBaaaleaacqWGQbGAaeqaaOGaeiilaWIaeeyAaKMaeeOzayMaeeiiaaIaemyAaKMaeeiiaaIaeeyyaeMaeeOBa4MaeeizaqMaeeiiaaIaemOAaOMaeeiiaaIaeeizaqMaeeyAaKMaeeOzayMaeeOzayMaeeyzauMaeeOCaiNaeeiiaaIaeeOyaiMaeeyEaKNaeeiiaaIaeeyyaeMaeeiiaaIaeeOBa4Maee4Ba8MaeeOBa4Maee4CamNaeeyEaKNaeeOBa4Maee4Ba8MaeeOBa4MaeeyEaKNaeeyBa0Maee4Ba8MaeeyDauNaee4CamNaeeiiaaIaeeiDaqNaeeOCaiNaeeyyaeMaeeOBa4Maee4CamNaeeODayNaeeyzauMaeeOCaiNaee4CamNaeeyAaKMaee4Ba8MaeeOBa4gaeaqabeaacqaHjpWDcqaH6oWAdaWgaaWcbaGaeeOuaifabeaakiabec8aWnaaBaaaleaacqWGQbGAaeqaaOGaeiilaWIaeeyAaKMaeeOzayMaeeiiaaIaemyAaKMaeeiiaaIaeeyyaeMaeeOBa4MaeeizaqMaeeiiaaIaemOAaOMaeeiiaaIaeeizaqMaeeyAaKMaeeOzayMaeeOzayMaeeyzauMaeeOCaiNaeeiiaaIaeeOyaiMaeeyEaKNaeeiiaaIaeeyyaeMaeeiiaaIaeeOBa4Maee4Ba8MaeeOBa4Maee4CamNaeeyEaKNaeeOBa4Maee4Ba8MaeeOBa4MaeeyEaKNaeeyBa0Maee4Ba8MaeeyDauNaee4CamNaeeiiaaIaeeiDaqNaeeOCaiNaeeyyaeMaeeOBa4Maee4CamNaeeyAaKMaeeiDaqNaeeyAaKMaee4Ba8MaeeOBa4MaeeiiaaIaeeOyaiMaeeyzauMaeeiDaqNaee4DaCNaeeyzauMaeeyzauMaeeOBa4MaeeiiaaIaeeiCaaNaeeyDauNaeeOCaiNaeeyAaKMaeeOBa4MaeeyzauMaee4CamhabaGaeqyYdCNaeqOUdS2aaSbaaSqaaiabbMfazbqabaGccqaHapaCdaWgaaWcbaGaemOAaOgabeaakiabcYcaSiabbMgaPjabbAgaMjabbccaGiabdMgaPjabbccaGiabbggaHjabb6gaUjabbsgaKjabbccaGiabdQgaQjabbccaGiabbsgaKjabbMgaPjabbAgaMjabbAgaMjabbwgaLjabbkhaYjabbccaGiabbkgaIjabbMha5jabbccaGiabbggaHjabbccaGiabb6gaUjabb+gaVjabb6gaUjabbohaZjabbMha5jabb6gaUjabb+gaVjabb6gaUjabbMha5jabb2gaTjabb+gaVjabbwha1jabbohaZjabbccaGiabbsha0jabbkhaYjabbggaHjabb6gaUjabbohaZjabbMgaPjabbsha0jabbMgaPjabb+gaVjabb6gaUjabbccaGiabbkgaIjabbwgaLjabbsha0jabbEha3jabbwgaLjabbwgaLjabb6gaUjabbccaGiabbchaWjabbMha5jabbkhaYjabbMgaPjabb2gaTjabbMgaPjabbsgaKjabbMgaPjabb6gaUjabbwgaLjabbohaZbaaaiaaxMaacaWLjaWaaeWaaeaacqaIXaqmaiaawIcacaGLPaaaaiaawUhaaaaa@588A@

### Estimating κ_R _and κ_Y_

Before generating the transition probability matrix, we need to estimate κ_R _and κ_Y_. In a similar way to YN's estimation of κ, we estimate four nucleotide frequencies (*g*_T_, *g*_C_, *g*_A_, *g*_G_), proportions of transitional differences between purines (*T*_R_) and between pyrimidines (*T*_Y_), and the proportion of transversional differences (*V*) from compared sequences. We calculate

a=log⁡(1−gR2gAgGTR−12gRV)b=log⁡(1−gY2gTgCTY−12gYV)c=log⁡(1−12gRgYV)     (2)
 MathType@MTEF@5@5@+=feaafiart1ev1aaatCvAUfKttLearuWrP9MDH5MBPbIqV92AaeXatLxBI9gBamXvP5wqSXMqHnxAJn0BKvguHDwzZbqegyvzYrwyUfgarqqtubsr4rNCHbGeaGqiA8vkIkVAFgIELiFeLkFeLk=iY=Hhbbf9v8qqaqFr0xc9pk0xbba9q8WqFfeaY=biLkVcLq=JHqVepeea0=as0db9vqpepesP0xe9Fve9Fve9GapdbaqaaeGacaGaaiaabeqaamqadiabaaGcbaqbaeaabiGaaaqaaiabdggaHjabg2da9iGbcYgaSjabc+gaVjabcEgaNjabcIcaOiabigdaXiabgkHiTmaalaaabaGaem4zaC2aaSbaaSqaaiabdkfasbqabaaakeaacqaIYaGmcqWGNbWzdaWgaaWcbaGaemyqaeeabeaakiabdEgaNnaaBaaaleaacqWGhbWraeqaaaaakiabdsfaunaaBaaaleaacqqGsbGuaeqaaOGaeyOeI0YaaSaaaeaacqaIXaqmaeaacqaIYaGmcqWGNbWzdaWgaaWcbaGaemOuaifabeaaaaGccqWGwbGvcqGGPaqkaeaacqWGIbGycqGH9aqpcyGGSbaBcqGGVbWBcqGGNbWzcqGGOaakcqaIXaqmcqGHsisldaWcaaqaaiabdEgaNnaaBaaaleaacqWGzbqwaeqaaaGcbaGaeGOmaiJaem4zaC2aaSbaaSqaaiabdsfaubqabaGccqWGNbWzdaWgaaWcbaGaem4qameabeaaaaGccqWGubavdaWgaaWcbaGaeeywaKfabeaakiabgkHiTmaalaaabaGaeGymaedabaGaeGOmaiJaem4zaC2aaSbaaSqaaiabdMfazbqabaaaaOGaemOvayLaeiykaKcabaGaem4yamMaeyypa0JagiiBaWMaei4Ba8Maei4zaCMaeiikaGIaeGymaeJaeyOeI0YaaSaaaeaacqaIXaqmaeaacqaIYaGmcqWGNbWzdaWgaaWcbaGaemOuaifabeaakiabdEgaNnaaBaaaleaacqWGzbqwaeqaaaaakiabdAfawjabcMcaPaqaaaaacaWLjaGaaCzcamaabmaabaGaeGOmaidacaGLOaGaayzkaaaaaa@8C5A@

where *g*_R _= *g*_A _+ *g*_G _and *g*_Y _= *g*_T _+ *g*_C_. Then we use equation 3 to estimate κ_R _and κ_Y_.

κR=a−gY×cgR×cκY−b−gR×cgY×c     (3)
 MathType@MTEF@5@5@+=feaafiart1ev1aaatCvAUfKttLearuWrP9MDH5MBPbIqV92AaeXatLxBI9gBamXvP5wqSXMqHnxAJn0BKvguHDwzZbqegyvzYrwyUfgarqqtubsr4rNCHbGeaGqiA8vkIkVAFgIELiFeLkFeLk=iY=Hhbbf9v8qqaqFr0xc9pk0xbba9q8WqFfeaY=biLkVcLq=JHqVepeea0=as0db9vqpepesP0xe9Fve9Fve9GapdbaqaaeGacaGaaiaabeqaamqadiabaaGcbaqbaeqabeGaaaqaaiabeQ7aRnaaBaaaleaacqqGsbGuaeqaaOGaeyypa0ZaaSaaaeaacqWGHbqycqGHsislcqWGNbWzdaWgaaWcbaGaemywaKfabeaakiabgEna0kabdogaJbqaaiabdEgaNnaaBaaaleaacqWGsbGuaeqaaOGaey41aqRaem4yamgaaaqaaiabeQ7aRnaaBaaaleaacqqGzbqwaeqaaOGaeyOeI0YaaSaaaeaacqWGIbGycqGHsislcqWGNbWzdaWgaaWcbaGaemOuaifabeaakiabgEna0kabdogaJbqaaiabdEgaNnaaBaaaleaacqWGzbqwaeqaaOGaey41aqRaem4yamgaaaaacaWLjaGaaCzcamaabmaabaGaeG4mamdacaGLOaGaayzkaaaaaa@660E@

d=−2gAgGgRa−2gTgCgYb−2(gRgY−gAgGgYgR−gTgCgRgY)c     (4)
 MathType@MTEF@5@5@+=feaafiart1ev1aaatCvAUfKttLearuWrP9MDH5MBPbIqV92AaeXatLxBI9gBamXvP5wqSXMqHnxAJn0BKvguHDwzZbqegyvzYrwyUfgarqqtubsr4rNCHbGeaGqiA8vkIkVAFgIELiFeLkFeLk=iY=Hhbbf9v8qqaqFr0xc9pk0xbba9q8WqFfeaY=biLkVcLq=JHqVepeea0=as0db9vqpepesP0xe9Fve9Fve9GapdbaqaaeGacaGaaiaabeqaamqadiabaaGcbaGaemizaqMaeyypa0JaeyOeI0YaaSaaaeaacqaIYaGmcqWGNbWzdaWgaaWcbaGaemyqaeeabeaakiabdEgaNnaaBaaaleaacqWGhbWraeqaaaGcbaGaem4zaC2aaSbaaSqaaiabdkfasbqabaaaaOGaemyyaeMaeyOeI0YaaSaaaeaacqaIYaGmcqWGNbWzdaWgaaWcbaGaemivaqfabeaakiabdEgaNnaaBaaaleaacqWGdbWqaeqaaaGcbaGaem4zaC2aaSbaaSqaaiabdMfazbqabaaaaOGaemOyaiMaeyOeI0IaeGOmaiJaeiikaGIaem4zaC2aaSbaaSqaaiabdkfasbqabaGccqWGNbWzdaWgaaWcbaGaemywaKfabeaakiabgkHiTmaalaaabaGaem4zaC2aaSbaaSqaaiabdgeabbqabaGccqWGNbWzdaWgaaWcbaGaem4raCeabeaakiabdEgaNnaaBaaaleaacqWGzbqwaeqaaaGcbaGaem4zaC2aaSbaaSqaaiabdkfasbqabaaaaOGaeyOeI0YaaSaaaeaacqWGNbWzdaWgaaWcbaGaemivaqfabeaakiabdEgaNnaaBaaaleaacqWGdbWqaeqaaOGaem4zaC2aaSbaaSqaaiabdkfasbqabaaakeaacqWGNbWzdaWgaaWcbaGaemywaKfabeaaaaGccqGGPaqkcqWGJbWycaWLjaGaaCzcamaabmaabaGaeGinaqdacaGLOaGaayzkaaaaaa@7B89@

The detailed procedures for deducing κ_R _and κ_Y _were summarized in the additional file 1. We also made other modifications accordingly, such as using κ_R _and κ_Y _to estimate S and N, generating relevant transition probability matrix (equation 1), considering different transitional evolution pathways to count S_d _and N_d_, and correcting for multiple substitutions when estimating Ka and Ks (equation 4; [22]).

## Authors' contributions

ZZ designed and programmed this new method, and drafted the manuscript. JL carried out computer simulations and generated sequence datasets. JY supervised the research and revised the manuscript. All authors read and approved the final manuscript.

## Supplementary Material

Additional File 1 Differences between the HKY and Tamura-Nei models and the detailed derivations of κ_R _and κ_Y_. This file contains two sections: section I shows the differences between the HKY and Tamura-Nei models and section II details the procedures for deducing κ_R _and κ_Y_. Click here for file
