# Supplementary material for: Computing Ka and Ks with a consideration of unequal transitional substitutions
Source: BMC Evol Biol. 2006 Jun 2;6:44. doi: 10.1186/1471-2148-6-44 (PMC1552089; doi:10.1186/1471-2148-6-44)
Supplement: Additional File 1 — Differences between the HKY and Tamura-Nei models and the detailed derivations of κR and κY. This file contains two sections: section I shows the differences between the HKY and Tamura-Nei models and section II details the procedures for deducing κR and κY. [file 1471-2148-6-44-S1.pdf]

Supplementary information

Computing Ka and Ks with a consideration of unequal transitional substitutions

Zhang Zhang[1,2,3#], Jun Li[2#], Jun Yu[1,2,4]*

1. Institute of Computing Technology, Chinese Academy of Sciences, Beijing 100080, China
2. Beijing Genomics Institute, Chinese Academy of Sciences, Beijing 101300, China
3. Graduate School of Chinese Academy of Sciences, Beijing 100039, China
4. James D. Watson Institute of Genome Sciences of Zhejiang University, Hangzhou Genomics Institute, Key Laboratory of Genomic Bioinformatics of Zhejiang Province, Hangzhou 310007, China

# These authors contributed equally to this work.
* To whom correspondence should be addressed.

1 Differences between the HKY and the Tamura-Nei Models

Table S1 Nucleotide Substitution Models

	A	T	C	G		A	T	C	G
		HKY Model					Tamura-Nei Model		
A	–	βg_T	βg_C	αg_G	A	–	βg_T	βg_C	$\boldsymbol{\alpha_R g_G}$
T	βg_A	–	αg_C	βg_G	T	βg_A	–	$\boldsymbol{\alpha_Y g_C}$	βg_G
C	βg_A	αg_T	–	βg_G	C	βg_A	$\boldsymbol{\alpha_Y g_T}$	–	βg_G
G	αg_A	βg_T	βg_C	–	G	$\boldsymbol{\alpha_R g_A}$	βg_T	βg_C	–

Note: α, transitional rate; β, transversional rate; α_R, transitional rate between purines; α_Y, transitional rate between pyrimidines; g_N, frequencies of nucleotide N, where $N \in \{T, C, A, G\}$.

2 Derivation of κ_R and κ_Y

Let us derive the equations for estimating κ_R and κ_Y. Tamura and Nei (1993) used g_T, g_C, g_A, and g_G to represent nucleotide frequencies for T, C, A, and G, respectively. They defined α_1, α_2, and β as transitional rates between purines and between pyrimidines, and transversional rate, respectively. They then derived the formulas (S1-S3) for the proportions of transitional differences between purines (P_1) and between pyrimidines (P_2) and of transversional differences (Q) over divergence time t [1]:

$$P_1 = \frac{2g_A g_G}{g_R}\{g_R + g_Y \exp(-2\beta t) - \exp[-2(g_R\alpha_1 + g_Y\beta)t]\} \tag{S1}$$

$$P_2 = \frac{2g_T g_C}{g_Y}\{g_Y + g_R \exp(-2\beta t) - \exp[-2(g_Y\alpha_2 + g_R\beta)t]\} \tag{S2}$$

$$Q = 2g_R g_Y[1 - \exp(-2\beta t)] \quad (S3)$$

where $g_R = g_A + g_G$ and $g_Y = g_T + g_C$.

Since P_1, P_2 and Q are estimable from sequence comparisons [1] and the observed number of substitutions underestimates the real number of substitutions as sequences diverge over time t, we need to calculate the real numbers of P_1, P_2 and Q, denoted as P_1', P_2' and Q', respectively. According to the Tamura-Nei Model, the formulas of P_1', P_2' and Q' are

$$P_1' = 4g_A g_G \alpha_1 t \quad (S4)$$

$$P_2' = 4g_T g_C \alpha_2 t \quad (S5)$$

$$Q' = 4g_R g_Y \beta t \quad (S6)$$

From S4–S6, we can derive the formulas for κ_R (S7) and κ_Y (S8).

$$\kappa_R = \alpha_1 / \beta = \frac{g_R g_Y P_1'}{g_A g_G Q'} \quad (S7)$$

$$\kappa_Y = \alpha_2 / \beta = \frac{g_R g_Y P_2'}{g_T g_C Q'} \quad (S8)$$

Since g_T, g_C, g_A and g_G can be estimated from compared sequences, therefore, the question now is "how to estimate P_1', P_2' and Q'". Due to the fact that the real number is often considered as a function of the observed number, we use S1–S3 and S4–S6 and obtain the formulas S9–S11 for P_1', P_2' and Q', respectively.

$$P_1' = \frac{2g_A g_G}{g_R}[g_Y \log(1 - \frac{1}{2g_R g_Y}Q) - \log(1 - \frac{g_R}{2g_A g_G}P_1 - \frac{1}{2g_R}Q)] \quad (S9)$$

$$P_2' = \frac{2g_T g_C}{g_Y}[g_R \log(1 - \frac{1}{2g_R g_Y}Q) - \log(1 - \frac{g_Y}{2g_T g_C}P_2 - \frac{1}{2g_Y}Q)] \quad (S10)$$

$$Q' = -2g_R g_Y \log(1 - \frac{1}{2g_R g_Y}Q) \quad (S11)$$

Now, we can obtain the formulas of κ_R and κ_Y as follows.

$$\kappa_R = \alpha_1 / \beta = \frac{g_R g_Y P_1'}{g_A g_G Q'} = \frac{\log(1 - \frac{g_R}{2g_A g_G}P_1 - \frac{1}{2g_R}Q) - g_Y \log(1 - \frac{1}{2g_R g_Y}Q)}{g_R \log(1 - \frac{1}{2g_R g_Y}Q)} \quad (S12)$$

$$\kappa_Y = \alpha_2 / \beta = \frac{g_R g_Y P_2'}{g_T g_C Q'} = \frac{\log(1 - \frac{g_Y}{2g_T g_C}P_2 - \frac{1}{2g_Y}Q) - g_R \log(1 - \frac{1}{2g_R g_Y}Q)}{g_Y \log(1 - \frac{1}{2g_R g_Y}Q)} \quad (S13)$$

In order to conveniently remember the meanings of P_1, P_2 and Q, in our main manuscript we rename them as T_R, T_Y, V, respectively. Hence, the formulas for estimating κ_R and κ_Y are as follows.

$$a = \log(1 - \frac{g_R}{2g_A g_G} T_R - \frac{1}{2g_R} V) \qquad b = \log(1 - \frac{g_Y}{2g_T g_C} T_Y - \frac{1}{2g_Y} V)$$

$$c = \log(1 - \frac{1}{2g_R g_Y} V) \tag{S14}$$

$$\kappa_R = \frac{a - g_Y \times c}{g_R \times c} \qquad \kappa_Y = \frac{b - g_R \times c}{g_Y \times c} \tag{S15}$$

Reference

1. Tamura K, Nei M: **Estimation of the number of nucleotide substitutions in the control region of mitochondrial DNA in humans and chimpanzees**. *Mol Biol Evol* 1993, **10**(3):512-526.
